# Pyrroloquinoline Quinone Inhibits Rotenone-Induced Microglia Inflammation by Enhancing Autophagy

**DOI:** 10.3390/molecules25194359

**Published:** 2020-09-23

**Authors:** Qi Zhang, Jing Zhou, Mi Shen, Hui Xu, Shu Yu, Qiong Cheng, Fei Ding

**Affiliations:** 1 Key Laboratory of Neuroregeneration of Jiangsu and Ministry of Education, Co-Innovation Center of Neuroregeneration, Nantong University, 19 Qixiu Road, Nantong 226001, China; zhangqi@ntu.edu.cn (Q.Z.); zhoujing961027@163.com (J.Z.); will21@ntu.edu.cn (M.S.); 13862964186@163.com (H.X.); yushu@ntu.edu.cn (S.Y.); 2 Jiangsu Clinical Medicine Center of Tissue Engineering and Nerve Injury Repair, Research Center of Clinical Medicine, Nantong 226001, China

**Keywords:** pyrroloquinoline quinone (PQQ), rotenone, microglia, neuroinflammation, autophagy

## Abstract

Neuroinflammation is a feature common to neurodegenerative diseases, such as Parkinson’s disease (PD), which might be responsive to therapeutic intervention. Rotenone has been widely used to establish PD models by inducing mitochondrial dysfunction and inflammation. Our previous studies have reported that pyrroloquinoline quinone (PQQ), a naturally occurring redox cofactor, could prevent mitochondrial dysfunction in rotenone induced PD models by regulating mitochondrial functions. In the present study, we aimed to investigate the effect of PQQ on neuroinflammation and the mechanism involved. BV2 microglia cells were pre-treated with PQQ followed by rotenone incubation. The data showed that PQQ did not affect the cell viability of BV2 cells treated with rotenone, while the conditioned medium (CM) of BV2 cells pre-treated with PQQ significantly increased cell viability of SH-SY5Y cells. In rotenone-treated BV2 cells, PQQ dose-dependently decreased lactate dehydrogenase (LDH) release and suppressed the up-regulation of pro-inflammation factors, such as interleukin-1β (IL-1β), IL-6 and tumor necrosis factor-α (TNF-α) in the cultured media, as well as nitric oxide (NO) release induced by rotenone. PQQ pretreatment also increased the ratio of LC3-II/LC3-I and expression of Atg5 in BV2 cells stimulated with rotenone. Additionally, the autophagosome observed by transmission electron microscopy (TEM) and co-localization of mitochondria with lysosomes indicated that mitophagy was induced by PQQ in rotenone-injured BV2 cells, and the PINK1/parkin mediated mitophagy pathway was regulated by PQQ. Further, autophagy inhibitor, 3-methyladenine (3-MA), partially abolished the neuroprotective effect of PQQ and attenuated the inhibition of inflammation with PQQ pretreatment. Taken together, our data extend our understanding of the neuroprotective effect of PQQ against rotenone-induced injury and provide evidence that autophagy enhancement might be a novel therapeutic strategy for PD treatment.

## 1. Introduction

Parkinson’s disease (PD) is a common neurodegenerative disease in the aged population, characterized with the dopaminergic neuron loss in substantia nigra pars compacta (SNpc) and the presence of Lewy bodies (LB). Neuroinflammation is considered as one of the pathological mechanisms in neurodegenerative diseases, including PD [1]. In the central nervous system (CNS), microglia cells play an important role in innate immunity and produce most of the pro-inflammatory factors in the human brain [2,3]. Activated microglia cells mediate inflammation by producing pro-inflammatory mediators, including interleukin-1β (IL-1β), IL-6, tumor necrosis factor-α (TNF-α) and nitric oxide (NO) [4]. The factors that can affect microglia activation might display neuroprotection in neurodegenerative diseases.

Pyrroloquinoline quinone (PQQ), a redox cofactor, was reported to attenuate microglia activation in lipopolysaccharide (LPS) treated mice and protect primary cortical neurons against neurotoxicity [5]. Our previous research also indicated that PQQ could regulate mitochondrial functions and antagonize rotenone induced neuronal injury both in vitro and in vivo [6,7,8], which might be a potential candidate for PD treatment. Rotenone is a widely used pesticide for establishing PD models [9]. The pathological hallmarks of PD can be reproduced by rotenone injection, both systemically and regionally [10,11]. Rotenone functions as a mitochondrial complex I inhibitor, which can easily cross the blood-brain barrier (BBB) to induce mitochondrial dysfunction, leading to oxidative stress, energy dysmetabolism and neuroinflammation [12,13]. In addition, rotenone can induce α-synuclein accumulation, as well as activation of astrocytes and microglia to mediate inflammatory responses [14]. However, whether PQQ could affect the microglia activation and regulate inflammation in rotenone-induced PD models has not been investigated.

Impaired autophagy is associated with neurodegenerative diseases and defects arise at different stages of the autophagy pathway [15]. Recent studies have proved that autophagy and innate immunity are closely related [16,17]. Rifampicin, a classic antibiotic, was reported to play protective effect against rotenone-induced microglia inflammation, and this effect was partially mediated by autophagy enhancement [13]. Autophagy is an evolutionarily conserved process, through which the damaged organelles and protein aggregates can be eliminated during stress and is necessary for maintaining homeostasis [18]. Damaged mitochondria is selectively eliminated through mitochondria autophagy (which is also known as mitophagy) [19] and the PINK1/parkin pathway is involved in mediating mitophagy process and selectively degradation of impaired mitochondria [20]. The modulation of autophagy is responsible for inflammatory processes in many diseases through regulating pro-inflammatory factors production [21]. Our previous study indicated that Akt/mTOR pathway activation might induce autophagy and protect against rotenone-induced SH-SY5Y cells injury [22]. Additionally, PI3K/Akt pathway can be activated by PQQ pretreatment in cultured midbrain neurons [6]. This research suggested that PQQ might be a promising candidate for PD treatment and regulation of microglia activation and autophagy might play important roles in this process.

In the present work, we aimed to investigate whether PQQ could attenuate rotenone-induced microglia inflammation via promoting autophagy. We demonstrated that PQQ exhibited protective effect against microglia-mediated cell death by inhibiting inflammation. Our data also suggested that enhancing autophagy by PQQ contributed to the regulation of inflammation and neuroprotective effects. The present study extended our previous work in investigating the molecular mechanisms involved in the neuroprotective effect of PQQ in rotenone-induced PD model.

## 2. Results

### 2.1. PQQ Exhibited Protective Effect against Microglia-Mediated SH-SY5Y Cell Death

**Figure 1:**
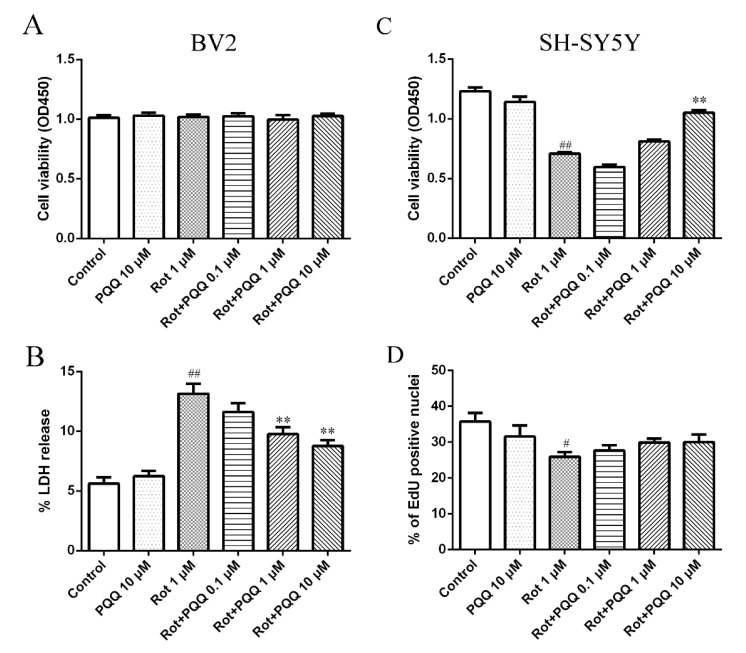
Pyrroloquinoline quinone (PQQ) rescued rotenone-induced microglia-mediated cell death. BV2 microglia cells were pre-treated with different doses (0.1, 1, 10 μM) of PQQ for 2 h before rotenone (1 μM) incubation for another 12 h. (**A**) Cell viability was measured with CCK-8 assay. No significant changes were observed in any group. (**B**) Lactate dehydrogenase (LDH) release in BV2 cells was measured. (**C**) The conditioned medium (CM) was collected from BV2 cells that were pre-treated with different doses (0.1, 1, 10 μM) of PQQ and rotenone for 12 h. SH-SY5Y cells were incubated with the CM from BV2 cells for 24 h and cell viability was measured with CCK-8 assay. (**D**) Cell proliferation of SH-SY5Y cells was measured with 5-ethynyl-2′-deoxyuridine (EdU) incorporation. The data were presented as the mean ± SEM from three or four independent experiments. For CCK-8 assay, 10 wells were included in one experiment. ** *p* < 0.01 vs. rot group (only treated with rotenone); # *p* < 0.05, ## *p* < 0.01 vs. control group (no rotenone or PQQ treatment).

We first examined whether PQQ affected cell viability of rotenone-treated BV2 microglia cells. The data of CCK-8 assay showed that 1 μM rotenone or 10 μM PQQ treatment alone did not influence BV2 cell viability. Neither did different concentrations of PQQ (0.1, 1, and 10 μM) pretreatment affect cell viability of BV2 cells with rotenone injury (Figure 1A). To detect whether the integrity of membrane was affected in such conditions, lactate dehydrogenase (LDH) release from BV2 cells was measured. Rotenone incubation increased the LDH release from BV2 cells, while PQQ pretreatment inhibited the LDH release at the concentration of 1 μM and 10 μM (Figure 1B). A previous study indicated that rotenone could induce pro-inflammatory factors release in BV2 cells, which might be toxic to neuronal cells in the CNS [23]. To determine whether PQQ could affect microglia-mediated neuronal cell death, in vitro assays was performed to investigate the effect of conditioned medium (CM) from rotenone-treated BV2 cells with or without PQQ pretreatment on SH-SY5Y cells by cell viability measurement. The cell viability of SH-SY5Y cells incubated with the CM collected from BV2 cells treated with rotenone decreased significantly compared to the control group, showing a toxicity effect. However, incubation with the CM from BV2 cells that were pre-treated with PQQ, especially at the concentration of 10 μM, increased the cell viability of SH-SY5Y cells significantly (Figure 1C). To further detect whether the increased cell viability was due to the increased proliferative capacity, 5-ethynyl-2′-deoxyuridine (EdU) assay was applied and the data indicated that the decreased cell proliferation induced by rotenone could not be rescued by PQQ pretreatment (Figure 1D). These data suggested that PQQ pretreatment might deserve protective effect against microglia-mediated SH-SY5Y cell death and this effect was not caused by increased cell proliferative ability.

### 2.2. PQQ Suppressed Rotenone-Induced Inflammation in BV2 Microglia

**Figure 2:**
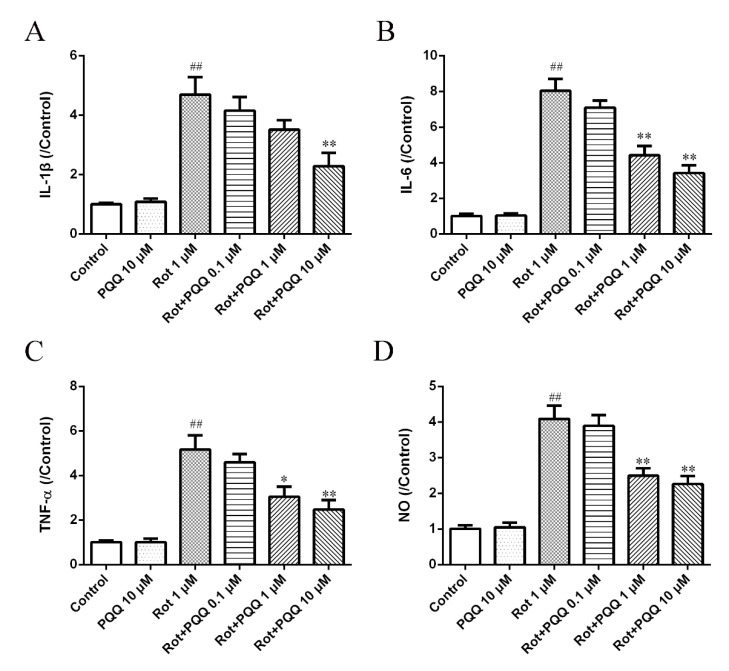
PQQ suppressed rotenone-induced pro-inflammatory cytokine and NO release in BV2 microglial cells. BV2 microglia cells were pre-treated with different doses (0.1, 1, 10 μM) of PQQ for 2 h before rotenone (1 μM) incubation for another 12 h. The levels of interleukin-1β (IL-1β) (**A**), IL-6 (**B**) and tumor necrosis factor-α ((TNF-α) (**C**) in the cultured media were measured by enzyme-linked immunosorbent assay (ELISA) assays. (**D**) Nitric oxide (NO) release in the cultured medium was measured. The data were presented as the mean ± SEM from four independent experiments. * *p* < 0.05, ** *p* < 0.01 vs. rot group; ## *p* < 0.01 vs. control group.

In order to examine whether PQQ pretreatment could regulate the inflammatory response of BV2 microglial cells with rotenone exposure, we stimulated BV2 cells with 1 μM rotenone after different concentrations of PQQ pretreatment for 2 h and the pro-inflammatory factors, including IL-1β, IL-6 and TNF-α in the cultured medium were detected by the enzyme-linked immunosorbent assay (ELISA) method. The production of IL-1β, IL-6 and TNF-α was significantly increased with rotenone stimulation (Figure 2A–C). Although PQQ alone did not affect the levels of pro-inflammatory factors, PQQ pretreatment significantly blocked the production of pro-inflammatory factors induced by rotenone, showing a dose-dependent manner (Figure 2A–C). Additionally, we also demonstrated that pretreatment with PQQ for 2 h could significantly reduce NO production in a concentration-dependent manner (Figure 2D), which was in consistent with a previous report that PQQ could attenuate NO release in BV2 microglia cells with LPS stimulation [5]. These data indicated that PQQ might suppress neuroinflammation in BV2 cells induced by rotenone.

### 2.3. PQQ Enhanced Autophagy in Rotenone-Injured BV2 Microglia

**Figure 3:**
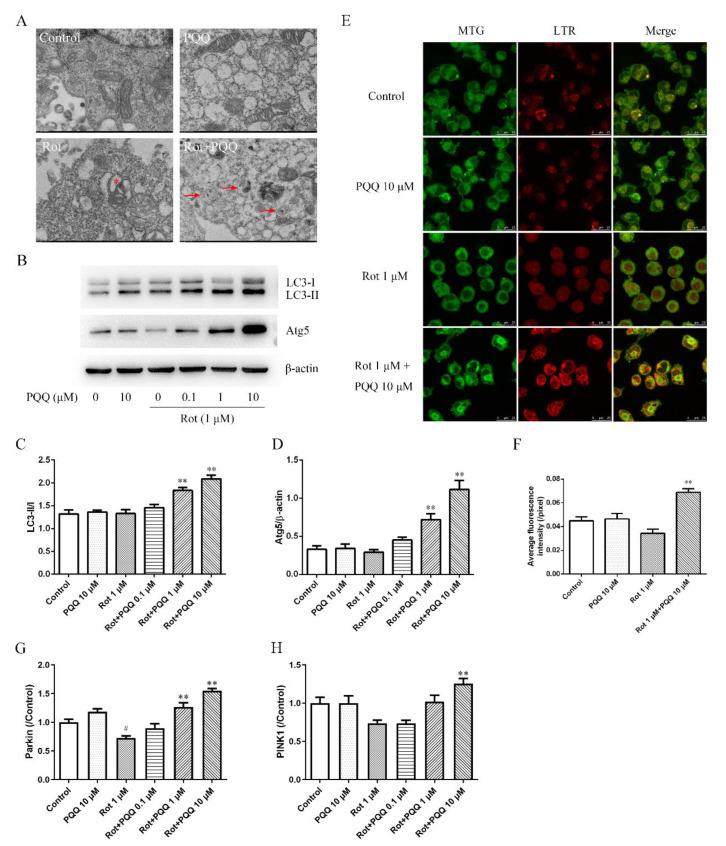
Autophagy was induced in BV2 microglia by PQQ. BV2 microglia cells were pre-treated with different doses of PQQ for 2 h before rotenone (1 μM) incubation for another 12 h. (**A**) Representative transmission electron microscopy (TEM) images of BV2 cells with different treatments as indicated. Asterisks indicate degenerated mitochondria. Arrows indicate autophagic vacuoles. Scale bar = 1 μm. (**B**) The protein expressions of LC3-I/LC3-II and autophagy related proteins 5 (Atg5) detected by Western blotting analysis. β-actin was used as a loading control. Quantification of the ratio of LC3-II/I (**C**) and the relative expression of Atg5 in BV2 cells (**D**). (**E**) Co-localization of mitochondria and lysosomes in BV2 cells detected by MitoTracker Green (MTG) and LysoTracker Red (LTR) staining. Cells with different treatments were incubated with 100 nM MTG and 100 nM LTR for 30 min. Images were acquired with a confocal microscope. Scale bar = 25 μm. (**F**) Average fluorescence intensity of MTG staining. Parkin (**G**) and PINK1 (**H**) mRNA expressions in BV2 cells measured by quantitative real-time reverse transcription PCR (qRT-PCR). The data were presented as the mean ± SEM from four independent experiments. ** *p* < 0.01 vs. rot group; # *p* < 0.05 vs. control group.

Autophagosome is considered as the marker of early stage in of autophagy. The images taken by transmission electron microscopy (TEM) showed ultrastructure changes of mitochondria and autophagic vacuoles in BV2 cells. In consistent with our previous study, rotenone induced pathological changes of mitochondria, including swelling and vacuolar degeneration in BV2 cells, while PQQ pretreatment attenuated the morphological changes and autophagic vacuoles containing swollen mitochondria appeared (Figure 3A). The ultrastructure images indicated that PQQ might induce autophagy in rotenone treated BV2 cells.

To further investigated the effect of PQQ pretreatment on autophagic dynamics, we detected the expression of LC3-I/LC3-II and autophagy related proteins 5 (Atg5) by Western blotting analysis. PQQ or rotenone alone did not affect the ratio of LC3-II to LC3-I, which was widely used as the indicator of autophagic activity. Both 1 μM and 10 μM PQQ significantly up-regulated the ratio of LC3-II to LC3-I in rotenone treated BV2 cells (Figure 3B,C). The expression of Atg5 was also up-regulated by 1 μM and 10 μM PQQ pretreatment (Figure 3B,D). MitoTracker Green (MTG) and LysoTracker Red (LTR) co-staining was also performed (Figure 3E). The fluorescent images showed that rotenone induced a slight decrease of fluorescent signals for mitochondria, while 10 μM PQQ pretreatment significantly increase the intensity for mitochondria and lysosomes (Figure 3F). Additionally, the co-localization of mitochondria and lysosomes increased significantly with PQQ pretreatment. Above data suggested that PQQ improved autophagy flux in rotenone induced BV2 cells.

To further determine whether PINK1/parkin pathway was affected with PQQ pretreatment, quantitative real-time reverse transcription PCR (qRT-PCR) was performed. The data showed that rotenone treatment caused a decrease in Parkin and PINK1 expression, although the change of PINK1 was not significant compared with the control group. However, PQQ pretreatment increased the mRNA expression of both Parkin and PINK1, indicating the activation of PINK1/parkin pathway (Figure 3G,H).

### 2.4. Autophagy Inhibitor Attenuated the Effect of PQQ on Rotenone-Induced Inflammation

**Figure 4:**
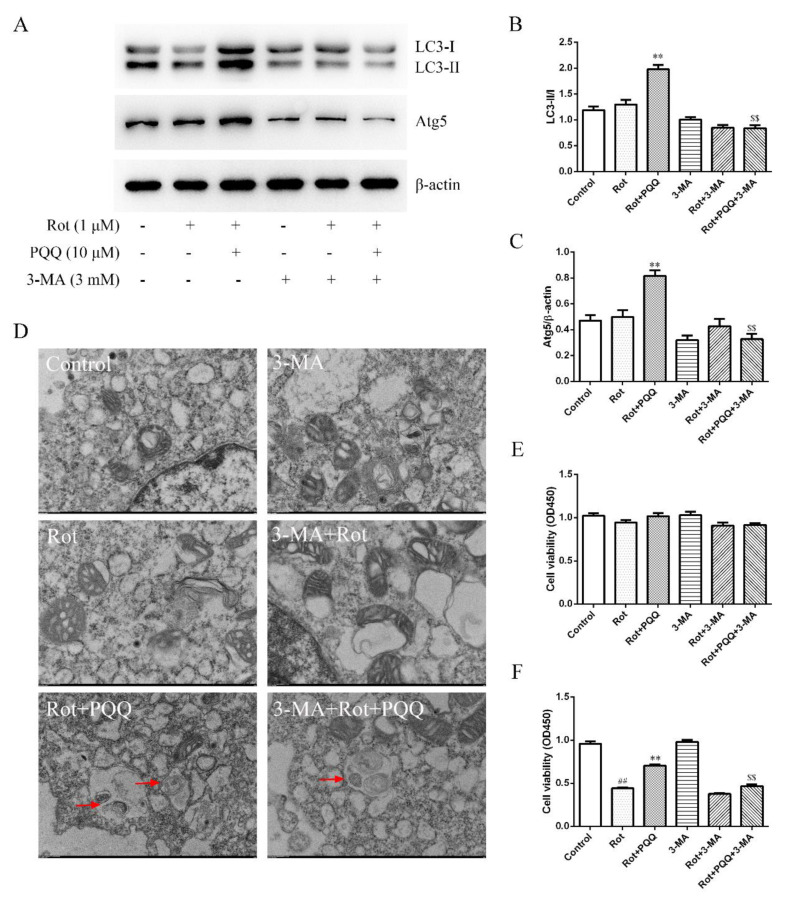
Inhibition of autophagy by 3-methyladenine (3-MA) reversed the protective effect of PQQ on rotenone-induced neurotoxicity. 3-MA was applied to the BV2 cells at the concentration of 3 mM 1 h before 10 μM PQQ pretreatment and 1 μM rotenone stimulation for 12 h. (**A**) Representative images of LC3-I/ LC3-II and Atg5 expressions detected by Western blotting analysis. β-actin was used as a loading control. Quantification of the ratio of LC3-II/I (**B**) and the relative expression of Atg5 in BV2 cells (**C**). The data were presented as the mean ± SEM from four independent experiments. (**D**) Representative TEM images of BV2 cells with different treatments as indicated. Arrows indicate autophagic vacuoles. Scale bar = 1 μm. (**E**) Cell viability of BV2 cells was measured with CCK-8 assay. (**F**) SH-SY5Y cells were incubated with the CM from BV2 cells with different treatments for 24 h and cell viability was measured with CCK-8 assay. The data were presented as the mean ± SEM from three or four independent experiments. For CCK-8 assay, 10 wells were included in one experiment. ** *p* < 0.01 vs. rot group; ## *p* < 0.01 vs. control group; $$ *p* < 0.01 vs. Rot+PQQ group.

3-methyladenine (3-MA) is a cell-permeable autophagy inhibitor, which protects cerebellar granule cells from apoptosis post serum or potassium deprivation. Here, we used 3-MA to clarify whether the enhancement of autophagy with PQQ pretreatment was responsible for the regulation of inflammation in rotenone-treated BV2 cells. The increased ratio of LC3-II to LC3-I and Atg5 expression with PQQ pretreatment was significantly inhibited by 3-MA incubation (Figure 4A–C). Autophagic vacuoles observed by TEM also decreased with 3-MA (Figure 4D). Although the cell viability of BV2 cells was not affected by 3-MA (Figure 4E), the increased cell viability of SH-SY5Y cells treated with CM from BV2 cells with rotenone and PQQ incubation was attenuated by 3-MA (Figure 4F). These data suggested that inhibition of autophagy attenuated the neuroprotective effect of PQQ.

**Figure 5:**
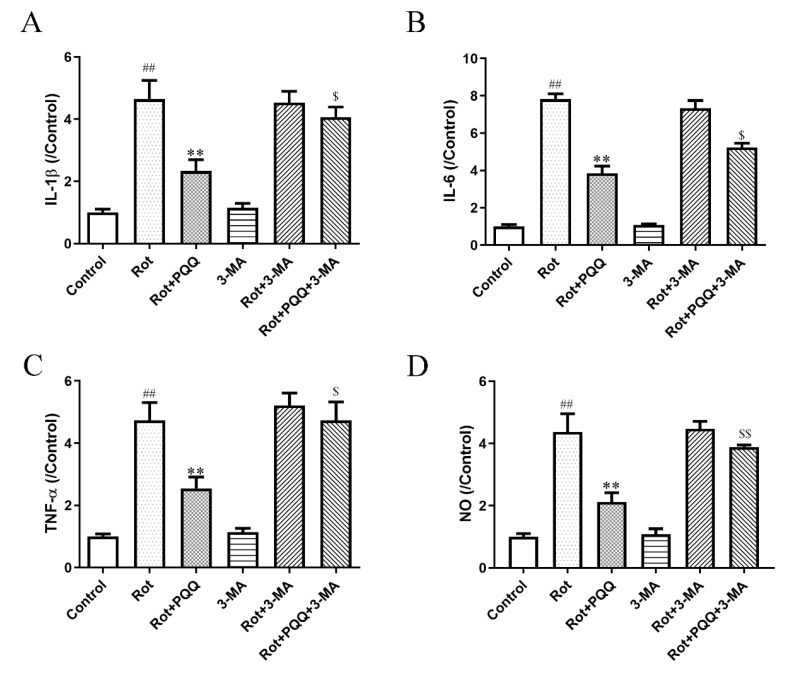
Autophagy inhibitor prevented the induction of pro-inflammatory cytokine and NO release induced by rotenone in BV2 microglial cells. 3-MA was applied to the BV2 cells at the concentration of 3 mM 1 h before 10 μM PQQ treatment and 1 μM rotenone stimulation for 12 h. The levels of IL-1β (**A**), IL-6 (**B**) and TNF-α (**C**) in the cultured media were measured by ELISA assays. (**D**) NO release in the cultured medium was measured. The data were presented as the mean ± SEM from four independent experiments. ** *p* < 0.01 vs. rot group; ## *p* < 0.01 vs. control group; $ *p* < 0.05, $$ *p* < 0.01 vs. Rot+PQQ group.

Last, we measured the effect of autophagy inhibition on microglia activation by pro-inflammatory factors. The suppressed production of IL-1β, IL-6, TNF-α and NO with PQQ pretreatment was also reversed by 3-MA (Figure 5A–D), indicating that autophagy inhibition attenuated the anti-inflammation effect of PQQ in rotenone simulated BV2 microglia activation.

## 3. Discussion

In this study, rotenone was used to induce neuroinflammation in BV2 microglia cells, and the effects of PQQ on inflammation and autophagy were investigated. Our data indicated that PQQ pretreatment in microglia could inhibit production of pro-inflammatory cytokines induced by rotenone and protect neuronal cells from microglia-mediated injury, which were achieved probably through autophagy enhancement.

The progressive degeneration of dopaminergic neurons and inflammatory processes are closely related in PD pathology [24]. The brains of both human PD patients and experimental animals share the common features of activated glial cells and increased pro-inflammatory factor levels [25], suggesting that neuroinflammation is involved in the pathology of PD. The accumulation of pro-inflammatory molecules is one of the mechanisms involved in neurodegeneration and eventually causes neurological diseases. Thus, treatment with anti-inflammatory drugs in the early stage among high-risk populations might be a potential therapeutic method for PD patients [26].

As a pesticide, rotenone is a mitochondrial complex I inhibitor, which is widely used to establish PD models in rodents. Chronic rotenone exposure might enhance oxidative stress to induce mitochondrial dysfunction and increase microglial proliferation and phagocytic activity, leading to neuronal loss [27,28]. Our previous research indicated that PQQ could protect dopaminergic neurons from cytotoxicity and prevented mitochondrial dysfunction in rotenone-induced PD models [6,29]. Here, we used rotenone to stimulate microglia activation in BV2 cells and the anti-inflammatory effect of PQQ and the mechanisms involved was investigated. Although the cell viability was not affected by rotenone in BV2 cells detected by CCK-8 measurement, the CM from microglia promoted damage of cultured neurons. LDH release assay indicated that rotenone could induce the loss of membrane integrity, which might favor the release of pro-inflammatory cytokines from the cytoplasm to induce microglial inflammation as detected by ELISA assays. On the other hand, PQQ pretreatment reduced the release of LDH and inhibited the production of pro-inflammatory cytokines, thus, attenuating the neurotoxicity of activated microglia. Our data demonstrated PQQ as a potential agent exerting neuroprotective effect through inhibition of pro-inflammatory mediators’ production in rotenone injury models.

Recently, the complex relationship between microglial autophagy and inflammation has been investigated [30]. Autophagy may balance the positive and negative effects of immunity in inflammatory diseases, and thereby plays protective effect [31]. Autophagy defects, caused by nutrient deprivation or autophagy proteins dysfunction, may increase activation of microglial and inflammation [32]. Recent studies revealed that the effect of rotenone on autophagy depended on the cellular system and the dosages used. Rotenone was reported to decrease autophagy, while increase mitophagy in neurons [33]. In our experiment, we did not observe significant effect of rotenone on the autophagy in rotenone-treated BV2 cells, and PQQ alone did not affect the autophagy. However, PQQ pretreatment increased autophagy in rotenone-treated BV2 cells, which was consistent with previous report that PQQ could inhibit the expression of pro-inflammatory mediators in microglia exposed to LPS and induce activation of the typical autophagy pathway [5]. The application of rotenone and PQQ together might lead to the combination of the subtoxic effects of rotenone and PQQ, which caused the activation of a cellular adaptive response, thus, inducing the autophagy in BV2 cells.

It was reported that autophagy could be induced by pro-inflammatory factors, such as IL-1β, IL-6, IL-17, TNF-α and IFN-γ. On the other hand, autophagy decreased the secretion of TNF-α, IL-1β and some other pro-inflammatory factors [34,35]. The enhanced inflammatory pathway activation regulated by autophagy deficiency was mediated by disruption of mitochondrial homeostasis, including the increase of mitochondrial ROS production and mitochondrial membrane permeability transition [36,37]. Our previous research indicated that PQQ could attenuate the decrease of mitochondrial membrane potential (MMP) and increase of ROS induced by rotenone in midbrain neurons [6]. PQQ could also induce the expression and translocation of redox-sensitive transcription factors including nuclear factor E2-related factor 2 (Nrf2) and activation of KEAP-1/Nrf2 pathway was responsible for the anti-inflammatory effect of PQQ by inhibiting a pro-inflammatory factor, nuclear factor kappa-B (NF-κB) [5,38,39,40,41]. Moreover, PQQ was reported to stimulate mitochondrial biogenesis and might be beneficial in diseases associated with mitochondrial dysfunction [42]. We also demonstrated that activation of Akt/mTOR signaling pathway in rotenone-injured SH-SY5Y cells could induce autophagy and protect the cells [22]. Together, this evidence suggested that rotenone might disrupt mitochondrial functions, thus, leading to autophagy defects and inflammation activation, while PQQ pretreatment could rescue the injury by regulating mitochondrial functions and oxidative stress.

3-MA is a widely used inhibitor of autophagy which blocks autophagosome formation through the PI3K inhibition [43]. Systemic application of 3-MA markedly improved anti-inflammatory effect by modulating autophagy [44]. Our data suggested that the application of 3-MA could partially reverse the inhibitory effect of PQQ pretreatment on the release of pro-inflammatory factors induced by rotenone, thus, further providing the evidence that autophagy might be involved in inflammation modulation by PQQ.

Mitophagy is a classic type of autophagy, which eliminates dysfunctional or damaged mitochondria. Clearance of damaged mitochondria inhibits the release of detrimental molecules which is necessary to protect cells from degeneration [45]. LC3 is an autophagosome marker which coats the autophagic vesicles and is required in mitophagy process [46]. The ratio of LC3-II to LC3-I (LC3-II/I) has been widely used as a marker in autophagic activity [47]. In this study, although no significant change in LC3-II/I ratio was detected with rotenone stimulation, the ratio of LC3-II/I was up-regulated by PQQ pretreatment in rotenone-treated BV2 cells. Combined with the formation of autophagic vacuoles observed by TEM, the data suggested that the autophagic activity was increased by PQQ. The formation of LC3-II requires the conjugation of LC3 to phosphatidylethanolamine and Atg5 is a key protein necessary for this process [48,49]. Atg5 was also detected to be up-regulated by PQQ pretreatment, further demonstrating the enhancement of autophagy. Moreover, PQQ pretreatment not only enhanced the fluorescence intensity of mitochondria stained with MTG and lysosome stained with LTR, but also promoted the co-localization of mitochondria and lysosomes. These data further supported that PQQ could promote mitophagy via lysosomal function.

The PINK1/Parkin pathway has been demonstrated to be related with mitophagy in recent studies [50,51]. PINK1 and Parkin accumulated on damaged mitochondria, promoted their segregation from the mitochondrial network [52]. The E3 ubiquitin ligase encoded by Parkin gene leads to the ubiquitination and degradation of mitochondrial proteins and the induction of mitophagy [53]. It was reported that Parkin could mediate neuroprotection through activation of NF-κB signaling [54] and Parkin knock out might contribute to dysregulated microglial responses [55]. Our study suggested that PQQ could up-regulate PINK1/Parkin pathway following rotenone injury, and this modulation might induce the mitophagy process in rotenone-treated BV2 cells.

## 4. Materials and Methods

### 4.1. Chemicals and Antibodies

Rotenone, PQQ, trypsin, monoclonal mouse anti-β-actin antibody were from Sigma (St. Louis, MO, USA). Monoclonal anti-LC3 mAb-HRP-DirecT was purchased from MBL (Woburn, MA, USA). Rabbit anti-Atg5 antibody, HRP-conjugated donkey anti-rabbit IgG and HRP-conjugated goat anti-mouse IgG were from Abcam (Cambridge, MA, USA). TRIzol reagent, MitoTracker Green (MTG) and LysoTracker Red (LTR) were obtained from Thermo Fisher Scientific (Carlsbad, CA, USA). ELISA kits for IL-1β, IL-6 and TNF-α, cell lysis buffer, protease inhibitor cocktail, BCA-based protein quantification kit, BeyoECL Plus and LDH Cytotoxicity Assay Kit were purchased from Beyotime (Shanghai, China). The Omniscript reverse transcription (RT) kit was purchased from Qiagen (Valencia, CA, USA). 5-ethynyl-2′-deoxyuridine (EdU) Labeling Kit was from Ribobio (Guangzhou, China). Fast EvaGreen qPCR Master Mix was purchased from Biotium (Hayward, CA, USA). Dulbecco’s Modified Eagle Medium (DMEM) and fetal bovine serum (FBS) were from Gibco (Grand Island, NY, USA). 3-methyladenine (3-MA) was from MCE (Monmouth Junction, NJ, USA).

### 4.2. Cell Culture and Treatment

Mouse BV2 microglia and SH-SY5Y cells were from American Tissue Type Culture Collection (ATCC) and cultured in DMEM supplemented with 10% FBS, 100 U/mL penicillin and 100 μg/mL streptomycin at 37 °C in a humidified atmosphere of 95% air and 5% CO_2_. SH-SY5Y is a human neuroblastoma cell line, which are widely used as in vitro models of neurodegenerative diseases, such as PD. Rotenone and PQQ was dissolved in DMSO at a concentration of 25 mM or dissolved in DMEM at a concentration of 4 mM for stock, respectively.

### 4.3. Cell Viability Measurement

BV-2 cells were plated in 96-well plates at a density of 5 × 10^5^ cells/mL before treatment. After attachment, the cells were pre-treated with different concentrations of PQQ (0.1, 1, 10 μM) for 2 h prior incubation with 1 μM rotenone for another 12 h. The cell viability was measured by CCK-8 method at the end of treatment. Briefly, WST-8 solution was added to the cultured cells in 96-well plates (10 μL/each well). After incubation at 37 °C for 1 h, the absorbance (OD) at 450 nm was measured with an ELx-800 microplate reader (Bio-Tek Inc., Winooski, VT, USA).

For the collection of the conditioned media (CM), the BV2 cells were plated in 6-well plates and treated with rotenone with or without PQQ as mentioned above for 12 h. SH-SY5Y cells plated in 96-well plates at a density of 5 × 10^5^ cells/mL were incubated with the CM from BV2 cells for 24 h before the cell viability was measured. 3-MA was applied to the BV2 cells 1 h before PQQ pretreatment at the concentration of 3 mM to inhibit autophagy as indicated.

### 4.4. Cell Toxicity Assay

BV-2 cells were treated as mentioned above. Cell toxicity was assessed by LDH release using the LDH Cytotoxicity Assay Kit according to the manufacturer’s protocol. In brief, LDH release regent was added to the cultured medium 1 h before detection, after which 120 μL of culture supernatant from each well was transferred to a new 96-well plate. 60 μL of LDH working solution was added to each well and incubated for 30 min in the dark at room temperature. The absorbance was measured by spectrophotometry at 490 nm with an ELx-800 microplate reader and the percentage of LDH release was calculated.

### 4.5. Cell Proliferation Assay

The proliferation capacity of SH-SY5Y cells with above treatment was evaluated by the EdU Labeling kit, according to the instructions. The cells were incubated with 50 mM EdU for 30 min, after which the cells were fixed and stained using anti-EdU solution, and the nuclei were stained with Hoechst 33342 dye. The images were taken using a Leica fluorescence microscope (Leica, Heidelberg, Germany). The percentage of EdU positive nuclei were calculated.

### 4.6. ELISA for Pro-Inflammatory Factors

BV2 microglia plated in 6-well plates were pre-treated with different concentrations of PQQ (0.1, 1, 10 μM) for 2 h and then treated with 1 μM rotenone for another 12 h. The CM was collected and the levels of IL-1β, IL-6 and TNF-α were measured by ELISA analysis, according to the manufacturer’s instructions.

### 4.7. NO Release Measurement

BV2 microglia were plated in 6-well plates were treated as mentioned above and the NO release in the supernatants of cultured medium was measured by Griess reagent assay kit, according to manufacturer’s instructions.

### 4.8. Detection of the Autophagic Degradation of Mitochondria (Mitophagy) by Immunofluorescence

BV2 cells plated in 24-well plates were pre-treated with PQQ for 2 h, followed by incubation with 1 μM rotenone for 12 h. Before harvest, the cells were washed with phenol free media and stained with 100 nM of MTG and 100 nM LTR for 30 min. Fluorescent images were taken using a laser confocal microscope (Leica, Heidelberg, Germany) and the average fluorescence intensity of Mitotracker staining was measured with ImageJ software (version 1.51, NIH, Bethesda, MD, USA).

### 4.9. Western Blotting Analysis

BV2 cells were treated with rotenone with or without PQQ as mentioned above and the total protein was extracted and quantified by BCA analysis, followed by electrophoresis separation on SDS-PAGE. After transferred to a PVDF membrane, the membrane was blocked with 5% non-fat dry milk in Tris-buffered saline (TBS, pH 7.4) and incubated with indicated antibodies at 4 °C overnight. After wash with TBS/T (TBS with 0.1% Tween 20), HRP-conjugated affinity purified donkey anti-rabbit IgG (1:5000) or goat anti-mouse (1:5000) was applied at room temperature for 2 h. The membrane was incubated with an ECL substrate and scanned with an imaging system (Tanon, Shanghai, China). For the detection of LC3-I and LC3-II, ECL substrate was directly applied to the membrane after washing off the first antibody. The data were analyzed with PDQuest 7.2.0 software (Bio-Rad, Hercules, CA, USA).

### 4.10. Transmission Electron Microscopy (TEM)

BV2 microglia cells were fixed with pre-cooled 2.5% glutaraldehyde and post-fixed with 1% osmium tetraoxide solution. After dehydrating stepwise in increasing concentrations of ethanol, the cells were embedded in Epon 812 epoxy resin and sectioned. The ultrathin sections were stained with lead citrate and uranyl acetate. The images were taken under a transmission electron microscope (JEOL Ltd., Tokyo, Japan).

### 4.11. qRT-PCR

Total RNA was extracted from BV2 cells with TRIzol regent and the mRNA was reverse transcribed into cDNA with an Omniscript RT kit, in accordance with the manufacturer’s instructions. cDNA samples were stored at −80 °C before use. qPCR was performed using the StepOne real-time PCR system (Applied Biosystems, Foster City, CA, USA). The reaction mixture was consisted of 5 μL of 2 × Fast EvaGreen Master Mix, 0.5 μL of forward primer, 0.5 μL of reverse primer, and 1 μL of cDNA. 18S rRNA was used as the internal control. The sequences of primers used for qPCR were shown in Table 1. The relative mRNA expression level of each gene was calculated by the comparative 2^−ΔΔCt^ method and normalized against 18S rRNA.

### 4.12. Statistical Analysis

Data were expressed as mean ± SEM. Statistical analysis was performed in GraphPad Prism 8.0 (GraphPad, San Diego, CA, USA). One-way analysis of variance (ANOVA) was and subsequent Tukey’s multiple comparisons test was used to compare the differences between groups. Statistically significant was set as *p* < 0.05.

## 5. Conclusions

In this study, we demonstrated that PQQ suppressed rotenone-induced microglial inflammation, and this effect was partially through enhancement of autophagy. In rotenone-treated BV2 cells, PQQ dose-dependently suppressed the up-regulation of inflammation factors and CM from BV2 cells pre-treated with PQQ protected SH-SY5Y cells against rotenone injury. Autophagy was induced by PQQ pretreatment in BV2 cells stimulated with rotenone. Additionally, the autophagy inhibitor 3-MA partially abolished the neuroprotective effect of PQQ and attenuated the inhibition of inflammation with PQQ pretreatment. Taken together, this research extended our understanding of the neuroprotective effect of PQQ against rotenone-induced injury and provided the evidence that autophagy enhancement might be a novel therapeutic strategy for PD.

## Figures and Tables

**Table 1:** Oligonucleotide sequences used in qRT-PCR analysis.

Gene	Primer Sequence (5′–3′)
Parkin	Forward-aaggggattgcgactcact Reverse-cttttgtccaccctgtaggc
PINK1	Forward-gcgaagccatcttaagcaaa Reverse-tgggaccatctctggatctt
18S rRNA	Forward-tagagggacaagtggcgttc Reverse-cgctgagccagtcagtgt
